# Utilization of maternal health care services and their determinants in Karnataka State, India

**DOI:** 10.1186/s12978-016-0138-8

**Published:** 2016-06-08

**Authors:** Marianne Vidler, Umesh Ramadurg, Umesh Charantimath, Geetanjali Katageri, Chandrashekhar Karadiguddi, Diane Sawchuck, Rahat Qureshi, Shafik Dharamsi, Anjali Joshi, Peter von Dadelszen, Richard Derman, Mrutyunjaya Bellad, Shivaprasad Goudar, Ashalata Mallapur, for the Community Level Interventions for Pre-eclampsia (CLIP) India Feasibility Working Group

**Affiliations:** Department of Obstetrics and Gynaecology, and the Child and Family Research Unit, University of British Columbia, Vancouver, BC Canada; Department of Community Medicine, S Nijalingappa Medical College, Bagalkot, Karnataka India; KLE University’s Jawaharlal Nehru Medical College, Belgaum, Karnataka India; Department of Obstetrics and Gynaecology, S Nijalingappa Medical College, Bagalkot, Karnataka India; Division of Women and Child Health, Aga Khan University, Karachi, Sindh Pakistan; Department of Family Practice, Faculty of Medicine, University of British Columbia, Vancouver, BC Canada; Department of Obstetrics, Christiana Care, Newark, DE USA

**Keywords:** Pregnancy, Rural population, Hypertension, Maternal Health Services, Postnatal care, Prenatal care, Pregnancy, High risk, India, Focus groups, Qualitative methods, Maternal Health Care Utilization

## Abstract

**Background:**

Karnataka State continues to have the highest rates of maternal mortality in south India at 144/100,000 live births, but lower than the national estimates of 190–220/100,000 live births. Various barriers exist to timely and appropriate utilization of services during pregnancy, childbirth and postpartum. This study aimed to describe the patterns and determinants of routine and emergency maternal health care utilization in rural Karnataka State, India.

**Methods:**

This study was conducted in Karnataka in 2012–2013. Purposive sampling was used to convene twenty three focus groups and twelve individual interviews with community and health system representatives: Auxiliary Nurse Midwives and Staff Nurses, Accredited Social Health Activists, community leaders, male decision-makers, female decision-makers, women of reproductive age, medical officers, private health care providers, senior health administrators, District health officers, and obstetricians. Local researchers familiar with the setting and language conducted all focus groups and interviews, these researchers were not known to community participants. All discussions were audio recorded, transcribed, and translated to English for analysis. A thematic analysis approach was taken utilizing an *a priori* thematic framework as well as inductive identification of themes.

**Results:**

Most women in the focus groups reported regular antenatal care attendance, for an average of four visits, and more often for high-risk pregnancies. Antenatal care was typically delivered at the periphery by non-specialised providers. Participants reported that sought was care women experienced danger signs of complications. Postpartum care was reportedly rare, and mainly sought for the purpose of neonatal care. Factors that influenced women’s care-seeking included their limited autonomy, poor access to and funding for transport for non-emergent conditions, perceived poor quality of health care facilities, and the costs of care.

**Conclusions:**

Rural south Indian communities reported regular use of health care services during pregnancy and for delivery. Uptake of maternity care services was attributed to new government programmes and increased availability of maternity services; nevertheless, some women delayed disclosure of pregnancy and first antenatal visit. Community-based initiatives should be enhanced to encourage early disclosure of pregnancies and to provide the community information regarding the importance of facility-based care. Health facility infrastructure in rural Karnataka should also be enhanced to ensure a consistent power supply and improved cleanliness on the wards.

**Trial registration:**

NCT01911494

**Electronic supplementary material:**

The online version of this article (doi:10.1186/s12978-016-0138-8) contains supplementary material, which is available to authorized users.

## Background

Health care utilization overall, and for maternal health specifically, has improved in India. Much of the progress made has been attributed to the National Rural Health Mission (NRHM) that has increased the number of community health workers and community level facilities, and resulted in more institutional deliveries [1, 2] (see Table 1). These community health workers (Accredited Social Health Activists, Auxiliary Nurse Midwives, Anganawadi worker, staff nurses) provide routine antenatal care in the home or government run centres (sub-centre, primary health centre, community health centre). Anganawadi workers provide the most basic health services, while Accredited Social Health Activists (ASHA) are facilitators for care, providing prevention and education.

**Table 1:** Site characteristics

	India	South India	Karnataka
Site characteristics			
Population	1,236,687		61,130,704 ^d^
# States	35		
Dominant religion	Hindu ^c^	Hindu ^c^	Hindu ^c^
Women’s literacy	55 % ^c^	68 % ^c^	58 % ^c^
Employment	36 % currently employed ^c^	41 % currently employed ^c^	40 % currently employed ^c^
Rural/urban	32 % urban ^a^		39 % urban ^d^
Fertility rate	2.8 ^c^	1.9 ^c^	2.1 ^c^
Maternal mortality ratio	178 per 100,000 live births ^b^	105 per 100,000 live births ^a^	144 per 100,000 live births ^a^
Maternal health care utilization			
Any ANC	76.4 % ^c^	94 % ^c^	89 % ^c^
≥4 ANC	48 % ^c^	89 % ^c^ (3+)	76 % ^c^ (3+)
Facility delivery (%)	39 % ^c^	79 % ^c^	65 % ^c^
Skilled attendant at delivery	47 % ^c^	84 % ^c^	70 % ^c^

^a^World Health Organization Country Profile: India 2012

^b^Office of the Registrar of India, 2013

^c^Demographic Health Survey 2013

^d^Rural Health Statistics in India 2012

The timing and frequency of visits in pregnancy and postpartum is not well known, there is also a paucity in the literature regarding the indications for which women and families feel necessary to seek care and the cultural beliefs guiding these decisions. Rural areas, such as the study areas of Belgaum and Bagalkot, have poorer coverage and access to maternal health care services and deserve targeted review.

Explanatory factors for under-utilization of maternal care services are numerous, and include: young maternal age; religion (Muslims in Karnataka were less likely to receive the recommended four antenatal visits); poor education; unskilled occupation; poverty; low caste; parity (higher birth order were less likely to access antenatal services); lack of autonomy; poor familial support; lack of access to transport; and the high cost of care and when it is accessed, its poor quality (such as shortages of supplies and staff, mistreatment by staff, or poor training or limited experience of staff) [1, 3–9]. These have been demonstrated overall and within Karnataka State, India specifically [1, 3].

As suboptimal levels of utilization are considered to contribute to poor maternal health, [1, 8, 10, 11], this study was designed to explore the patterns of maternal health care utilization in rural Karnataka, as well as to identify the barriers that must be addressed to raise levels of that utilization with the ultimate goal of improving maternal and perinatal outcomes. This study further contributes to the literature by providing many community and health system perspectives. Previous studies have focused on the perspectives of health care providers, pregnant women and at times husbands, seldom has research on this topic included the views of other important decision-makers, particularly mothers-in-law.

## Methods

### Study setting

**Fig. 1:**
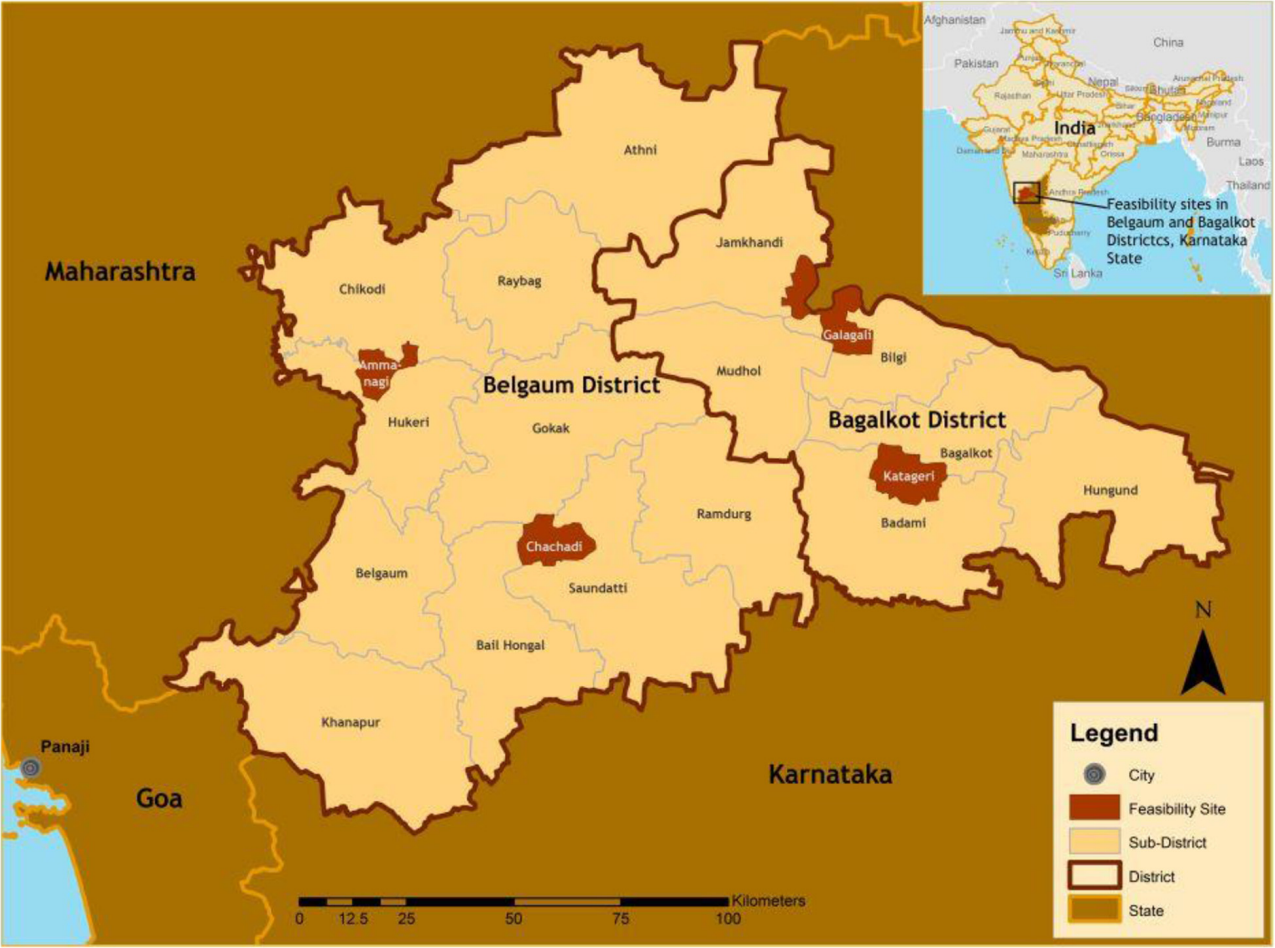
Map of study sites, Karnataka State, India

This qualitative study was conducted in two rural Districts of Karnataka State (Belgaum and Bagalkot) (Fig. 1) where women have higher birth order, lower maternal age, and are more likely to be illiterate than women in other southern states [3]. These differences reflect variable political commitment to maternal health, poor implementation of the NRHM, and decentralization of services [12].

This study was conducted as part of a feasibility study for a cluster randomized controlled trial of a community-based treatment package for pre-eclampsia administered by community health care workers in Karnataka State (NCT01911494) [13]. This formative research focused on exploration of the knowledge, attitudes and beliefs of community groups as well as facility assessments; the detailed methods are provided in [14].

### Data collection

Data were collected from January to March 2013 through focus group discussions and one-to-one interviews with community members and health care providers. Purposive sampling was used; focus group participants were recruited by invitation of primary health centre staff, some during routine antenatal visits. Each focus group was assembled separately by type of respondent with the exception of Auxiliary Nurse Midwives (ANM) and staff nurses who participated together. All focus groups were conducted in the local language, Kannada, at primary health centres which are familiar to participants and have minimal distractions. All concerned senior health administrators, private consultants, and obstetricians at teaching institutes were recruited for in-depth interviews. All interviews were conducted in English. Local researchers conducted interviews and focus groups, these facilitators have backgrounds in community or obstetric medicine and underwent qualitative research training prior to the commencement of the study. Interviewers were not known to community participants. However, some interviewees had prior interactions with interviewers, this was avoided when possible. Interview participants were provided a small compensation for work time lost. Focus groups discussions lasted on average 60–90 min, while interviews were 45–60 min.

Focus group and interview guides were semi-structured and designed to focus on maternity care utilization and the challenges to it, key areas included routine antenatal care, pregnancy complications or danger signs, delivery, and postpartum care, decision-making power, transport, and quality of services. All discussions were audio recorded, photographs and field notes were also taken. All recordings were reviewed to ensure accuracy; responses were then transcribed verbatim into English.

### Participants

A total of twenty-three focus groups were held with community health workers (ANMs and staff nurses) (*n* = 48), Accredited Social Health Activists (ASHA) (*n* = 53), community leaders (*n* = 27), male (*n* = 19) and female (*n* = 41) decision-makers, women of reproductive age (*n* = 132), and one with medical officers (*n* = 12) (Table 2). Twelve interviews with two of each of the following stakeholders: medical officers (each of whom is responsible for one primary health centre that usually serves as the entry point to the health system), private consultants, senior health administrators, District health officers, and four obstetricians from tertiary level health care centres.

**Table 2:** Characteristics of focus group participants

#	N participants	Age (yr)	Occupation	Child <5 yr	Pregnant	Education	Relationship to woman
		Median [range]	1. Housewife 2. Labourer 3. Employee 4. Self-employed 5. Other			1. No formal schooling, cannot read or write 2. No formal schooling, can read and write 3. Primary school incomplete 4. Primary school complete 5. Secondary school incomplete 6. Secondary school complete 7. Pre-university incomplete 8. Pre-university complete 9. University incomplete 10. University complete 11. Postgraduate 12. Don’t know	1. Husband 2. Father 3. Father-in-law 4. Mother-in-law 5. Mother 6. Other
Community leaders							
1	7	36 [31,48]	1 = (*N* = 1)	Not asked	Not asked	1 = (*N* = 1)	Not asked
			2 = (*N* = 6)			6 = (*N* = 1)	
						8 = (*N* = 2)	
						10 = (*N* = 3)	
2	10	36 [24,51]	1 = (*N* = 3)	Not asked	Not asked	1 = (*N* = 1)	Not asked
			4 = (*N* = 3)			3 = (*N* = 1)	
			5 = (*N* = 4)			4 = (*N* = 1)	
						5 = (*N* = 1)	
						6 = (*N* = 3)	
						8 = (*N* = 1)	
						9 = (*N* = 1)	
						10 = (*N* = 1)	
3	10	Not known	Not known	Not asked	Not asked	Not known	Not asked
Male decision-makers							
1	8	26 [18,57]	2 = (*N* = 4)	Not applicable	Not applicable	1 = (*N* = 4)	1 = (*N* = 4)
			3 = (*N* = 1)			3 = (*N* = 2)	3 = (*N* = 2)
			4 = (*N* = 1)			6 = (*N* = 2)	6 = (*N* = 2)
			5 = (*N* = 2)				
2	11	49 [33,59]	3 = (*N* = 2)	Not applicable	Not applicable	3 = (*N* = 4)	2 = (*N* = 4)
			5 = (*N* = 9)			5 = (*N* = 1)	3 = (*N* = 3)
						6 = (*N* = 1)	6 = (*N* = 4)
						10 = (*N* = 1)	
						12 = (*N* = 4)	
Female decision-makers							
1	10	45 [30,60]	1 = (*N* = 9)	Not applicable	Not applicable	1 = (*N* = 8)	4 = (*N* = 6)
			2 = (*N* = 1)			4 = (*N* = 2)	5 = (*N* = 2)
							6 = (*N* = 2)
2	18	45 [28,65]	1 = (*N* = 5)	Not applicable	Not applicable	1 = (*N* = 3)	Not known
			3 = (*N* = 1)			2 = (*N* = 1)	
			4 = (*N* = 1)			3 = (*N* = 1)	
			5 = (*N* = 11)			12 = (*N* = 13)	
3	13	48 [30,65]	1 = (*N* = 13)	Not applicable	Not applicable	3 = (*N* = 1)	4 = (*N* = 7)
						5 = (*N* = 1)	5 = (*N* = 1)
						12 = (*N* = 11)	6 (*N* = 5)
Women of reproductive age							
1	55	Not known	Not known	Not known	Not known	Not known	Not known
2	16	25 [20,30]	1 = (*N* = 16)	56 %	75 %	3 = (*N* = 3)	Not applicable
						4 = (*N* = 1)	
						5 = (*N* = 1)	
						6 = (*N* = 2)	
						8 = (*N* = 2)	
						10 = (*N* = 1)	
						12 = (*N* = 6)	
3	14	23 [18,30]	1 = (*N* = 9)	36 %	86 %	1 = (*N* = 3)	Not applicable
			2 = (*N* = 5)			3 = (*N* = 3)	
						4 = (*N* = 1)	
						5 = (*N* = 1)	
						6 = (*N* = 3)	
						8 = (*N* = 2)	
						10 = (*N* = 1)	
4	17	Not known	1 = (*N* = 17)	88 %	100 %	Not known	Not applicable
5	14	22 [18,58]	1 = (*N* = 12)	71 %	50 %	3 = (*N* = 3)	Not applicable
			3 = (*N* = 2)			5 = (*N* = 3)	
						6 = (*N* = 3)	
						10 = (*N* = 2)	
						12 = (*N* = 3)	
6	16	20 [19,26]	1 = (*N* = 16)	69 %	63 %	3 = (*N* = 7)	Not applicable
						4 = (*N* = 4)	
						5 = (*N* = 1)	
						6 = (*N* = 3)	
						8 = (*N* = 1)	
Auxiliary nurse midwives and staff nurses							
1	8	36 [24,56]	Not known	Not asked	Not asked	6 = (*N* = 3)	Not applicable
						8 = (*N* = 1)	
						10 = (*N* = 1)	
						11 = (*N* = 3)	
2	9	41 [23,58]	5 = (*N* = 9)	Not asked	Not asked	6 = (*N* = 2)	Not applicable
						7 = (*N* = 1)	
						8 = (*N* = 3)	
						10 = (*N* = 3)	
3	19	39 [25,58]	SN (*N* = 7)	Not asked	Not asked	Not known	Not applicable
			ANM (*N* = 10)				
4	12	30 [24,53]	SN (*N* = 4)	Not asked	Not asked	Not known	Not applicable
			ANM (*N* = 4)				
Accredited social health activists							
1	10	32 [26,36]	Not known	Not asked	Not asked	Not known	Not applicable
2	11	Not known	Not known	Not asked	Not asked	Not known	Not applicable
3	15	Not known	ASHA (*N* = 15)	Not asked	Not asked	Not known	Not applicable
4	17	33 [21,44]	ASHA (*N* = 17)	Not asked	Not asked	Not known	Not applicable
Medical officers							
1	15	Not known	Not asked	Not asked	Not asked	Not asked	Not applicable

Data saturation was judged to be sufficiently met in the planned number of focus groups and interviews, as preliminary analyses revealed that similar themes and findings were emerging repeatedly; therefore no additional data collection was done. Respondent characteristics within each stakeholder group were relatively homogeneous but there were some between group differences (Tables 2 and 3). All community health workers were actively providing care in the community at the time of data collection.

**Table 3:** Characteristics of interview participants

#	Stakeholder	Training	Level of care	Pregnancies/Week	Catchment pop	Pre-eclampsia/12 months
1	Medical officer	MBBS	Primary	50–60	19,000	5–6
2	Medical officer	MBBS	Primary	10–15	35,000	20
3	Private practitioner	MD OBG	Tertiary	40–50	200,000	50–100
4	Private practitioner	MBBS & MD OBG	Tertiary	280–300	300–400,000	20–25
5	Senior health administrator	MS General Surgery	*--*	*--*	1,950,000	*--*
6	Senior health administrator	MBBS & PG in OBG	*--*	*--*	4,800,000	*--*
7	District health officer	Speciality DGO	Secondary	200–250	25,000	12–25
8	District health officer	MBBS & Diploma OBG	Secondary	50–60	270,000	200–220
9	Obstetrician	MD OBG	Tertiary	250	Unknown	80–100
10	Obstetrician	MBBS & DGO & MD	Tertiary	200–300	300,000	500+
11	Obstetrician	MBBS & Diploma OBG	Tertiary	45	Unknown	150–200
12	Obstetrician	MD OBG	Tertiary	--	800–900,000	15–17 %

### Analysis

**Fig. 2:**
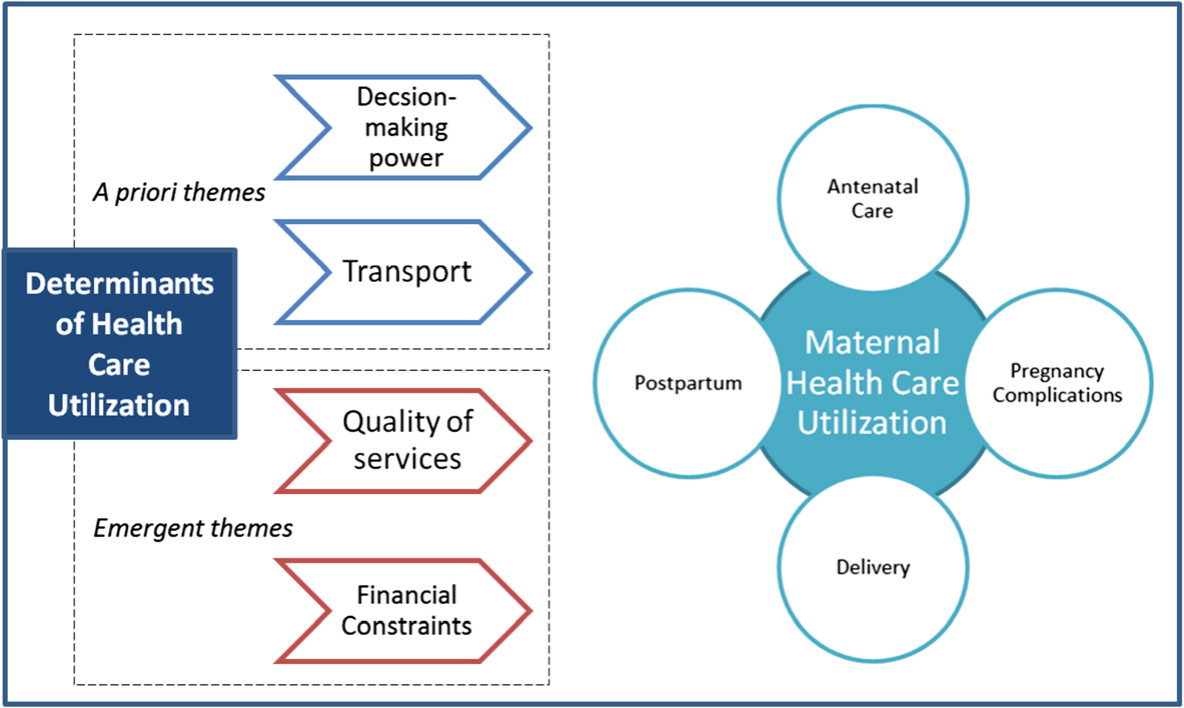
Thematic analysis structure

An *a priori* thematic framework was used as an initial guide and supplemented as novel themes emerged from the data (Fig. 2). English interview and focus group transcripts, after initial analysis and de-identification, were sent to the study co-ordinating office, University of British Columbia (UBC), where they were reviewed and coded by the study qualitative research manager (MV). Once coded, all transcripts were returned to the relevant local research teams at Jawaharlal Nehru Medical College (JNMC), Belgaum and S Nijalingappa Medical College (SNMC), Bagalkot. This stage was followed by a detailed review of the transcripts and preliminary analysis findings between the coder (MV) and in-country study co-ordinators (UC), focus group/interview facilitators and transcribers (GK, UR, SB, SB, CK), as well as principal investigators (MB, AM). This review included the identification of new and emergent themes as well as confirmation of the data interpretation. At the time of this review, any misinterpretations of the data were corrected after review of the original transcript and group consensus was reached. Themes were developed and grouped to reflect wider concepts; to provide insight into the subjective experiences of participants.

### Ethical approval

This study was approved by the ethics review committees at the University of British Columbia, Vancouver Canada (H12-00132) and KLE University, Belgaum India (MDC/IECHSR/2011-12).

## Results

### Maternal health care utilization

#### Antenatal care

The timing of the first antenatal care visit varies; according to providers there is now a trend to present earlier in gestation, by the end of the third month. Still, women described cultural beliefs that implore them to conceal pregnancies, this includes the belief that one should not cross a river early in pregnancy: “*It is a blind belief (superstition) [that it is best] not to disclose”* [ANM/staff nurse]. Identification of pregnancies may be limited by availability of pregnancy tests: *“Many times ANMs or ASHAs do not have a free supply of pregnancy kits”* [ANM/staff nurse]. Women do not systematically track their menstrual cycle, and have difficulty recalling their last menstrual period; as a result, asymptomatic women may not know they are pregnant. ASHAs attempt to track women of reproductive age in the community to identify their pregnancies early, but are challenged by women’s concealment.

Once the pregnancy is disclosed, women generally attend antenatal care. “*During pregnancy we visit for check-up”* [woman of reproductive age]. The frequency of visits varies significantly between respondents, with women reporting an average of four antenatal care (ANC) visits. More visits are reportedly made near term and for those at high-risk of complications. Very few reported coming for fewer than three visits. This is congruent with and attributed to government programs and recommendations: “*Nowadays antenatal visits are more because of incentives”* [medical officer]. Government programs guided by the National Rural Health Mission include cash incentives for antenatal care visits and institutional deliveries.

Antenatal care occurs at all levels of the health system (public and private). In these communities, there is no longer regular use of Dais (traditional birth attendants): “*No Dais working now, all that has decreased now”* [ANM/staff nurse]. Similarly, though many women use folk remedies in pregnancy, the use of traditional providers is rare – “*In older times there were many such people* [traditional healers]*”; “[pregnant women] are scared […] if they take such medicine […] and if something goes wrong”* [male decision-makers]; *“Nowadays [traditional medicines] are not used, they prefer hospital treatment”* [female decision-makers].

#### Complications

It was widely agreed that care should be sought if a woman is at high-risk of complications or if she displayed danger signs, such as eclampsia. The responses focused on what is recommended, which may not reflect the reality of practice: *“when a pregnant woman feels pain she should come to the hospital immediately”* [ANM/staff nurse]. Almost all women in the area delivered institutionally and reported using services for complications or emergencies. One woman described her feelings regarding accessing services for emergencies: “*[We] do not have any problem in going to hospital during emergency, if [we] are not feeling alright then only they go to the hospital”* [woman of reproductive age]. Generally, if complications are perceived to be minor, women access care at the primary health centre; however, if they are perceived as more serious, care is sought at a higher-level facility where specialists are available: *“If it is severe we may take her to the hospital”* [community leader]. Much of the discussion was related to pre-eclampsia and eclampsia as this was a topic of interest for researchers and probed directly: “*I had fits in the home so I went to the hospital”* [woman of reproductive age]. Bleeding and the absence of fetal movements were also commonly mentioned as reasons for care seeking at the higher facility: *“If pain in the abdomen is there, if the fetus does not move properly”* [woman of reproductive age].

#### Delivery

Many women stated that they visit the hospital at the time of delivery, supported by the high rates of facility deliveries: *“Nowadays women are all aware of medical care so they go to the hospital”* [male decision-maker]; *“During delivery time also we go, we have to go”* [woman of reproductive age]; *“Everyone goes to the hospital”* [woman of reproductive age]. Participants often attributed facility birth to government financial and material incentives, such as small remuneration and basic items (Madilu kit) for care of the newborn. An ANM/staff nurses in Bagalkot described the influence of these demand side initiatives: “*Women don’t deliver at home now at least for the sake of Madilu Kit*” [ANM/staff nurse].

#### Postpartum

Few respondents mentioned postpartum health care utilization, during discussion of ‘when to seek care related to childbirth’. When they did, care was usually sought for newborn care or feeding difficulties: “*If the baby cannot breastfeed”* [ANM/staff nurse]. Some community health care providers stated that women rarely accessed services after delivery until the infant requires immunizations: *“For immunization of the child”* [woman of reproductive age].

#### How providers encourage women to seek care

Health care providers have adopted a number of strategies to encourage women to better access services. Some providers stated that they offer to: accompany women to referral facilities, provide services in the home, and supply financial assistance or even free services. In addition, providers stated that they encourage women to access appropriate and timely facility services, particularly for delivery. This health care information is targeted at the entire family, particularly husbands and mothers-in-law, regarding available facilities and services, possible pregnancy complications, and the importance of antenatal care. One medical officer described the components of this education: “*If you get a check-up, what will happen, if you don’t get a check-up, that we will tell and convince and will make them to come to the hospital. Calling the mothers-in-law and whatever relations they will be having call them and explain the risks and everything and what happens in the regular ANC check up and take iron tablets”* [medical officer].

### Determinants of health care utilization in pregnancy and postpartum

#### Decision-making power

Respondents confirmed that the autonomy of women in patriarchal Indian communities remains limited. Respondents almost unanimously claimed that women have restricted decision-making power concerning their own health, and few mentioned that this may be changing: “*Nowadays [women] take their own decisions”* [female decision-maker]. Mothers-in-law and husbands hold the decision-making power, and they often resist facility-based care: *“The elders say why waste money in going to hospital, they can deliver at home without any problem”* [ASHA]; *“Sometimes elders in the family insist for home delivery”* [male decision-maker]. Stated reasons for this included the family’s history of successful home births, as described by many participants: “*I delivered 10 to 12 times without any problems, why she should go to the hospital for delivery?”* [ANM/staff nurse]; *“Elderly people don’t allow them to go to the hospital as they themselves delivered at home”* [ASHA]. As a result, women who are home alone must wait for a designated decision-maker before appropriate action can be considered: *“If nobody is home, she cannot go alone”* [ANM/staff nurse]. This lack of familial support was reported to be widespread and to prevent or delay care-seeking. At times, neighbours may stand in if the household decision-maker is unavailable: *“If family members have gone out […] and the pregnant woman is home alone and she starts getting pains, neighbours should get her to the hospital”* [ASHA].

#### Availability of transport

Communities in Karnataka reported awareness of ‘108’ - the robust ambulance system available free of charge for emergencies: *“If we have to go to Bagalkot they provide ambulance free of cost, previously it was not good, now it is good”* [community leader]. The challenges expressed related to transport for routine care. Women may need to hire private vehicles, but some villages do not have access to these as backup and some families cannot afford this. The price of private transport varies depending on the mode, time of day and distance, some state it can be as little as 100 Rupees (equivalent to $1.50 USD) and as high as 3,000 Rupees (equivalent to $47 USD). Many respondents stated that at the community level, funds are available for transport for those below the poverty line. Aside from availability and cost, access was described to be further complicated by the terrain, with narrow roads and villages at long distances from urban centres. These were stated to be barriers to seeking transport and care, particularly at night: *“Nobody will be there at night, in villages we don’t have access to transportation at night”* [community leader].

#### Quality of services and providers

Most respondents stated they were satisfied with the quality of services: “*All are happy about the quality”* [male decision-maker]. However, some complained about the poor maintenance of facilities as a barrier. Specific issues raised included lack of sufficient staffing and inadequate infrastructure, such as unreliable power and poor level of cleanliness: “*We need more rooms, good water, consistent electricity, and also tea and food should be given”* [woman of reproductive age]. Also, facilities are reportedly short of the necessary medical equipment, materials, lab services, blood, and medications.

The majority of participants stated that they had been well treated by health care staff: “*Our health care providers speak nicely and they see us calmly and solve any doubt we have, we are happy for that”* [woman of reproductive age]. Previous experiences were mentioned as influencing a woman’s service utilization in a subsequent pregnancy: “*Wherever they have faith, they will go; it is dependent on their belief. They would have gone repeatedly before and they have felt good at that time”* [community leader]. Trust and respect are essential qualities in a health care provider, according to women of reproductive age. Obstetricians themselves claim when women have trust in the doctor they will come to facility.

#### Financial constraints

Respondents indicated that the costs of health care inhibit women’s ability to access health care services: “*Fear of cost at facility makes them think twice”* [ANM/staff nurse]. These costs are related to transport, medications, services, lost wages, and food. Respondents reported that the cost of delivery ranges from 500Rs (equivalent to $8 USD) up to 100,000Rs (equivalent to $1,570 USD). The cost of delivery largely depends on the type of facility (government vs private) and the services provided (mode of delivery and associated complications). The community may provide financial support to women in need through women’s groups (Stree Shakti Sangh), Gram Panchayat (local self-government), and government programs. Those who qualify as below the poverty line are provided these subsidies and additional benefits to encourage appropriate care-seeking.

## Discussion

Several underlying factors have been identified to play important roles in maternal health care seeking: autonomy, access to transport, quality of facilities, incentive-based programs and poverty. It has been shown that women in rural areas present later in gestation which matches these findings [5, 15]. Previous research has shown that women’s autonomy (decision-making power related to her own health, buying household items, going to stay with family, and access to funds) is low in India which influences utilization of ANC, delivery and postpartum examination [16, 17]. Similarly, this study highlighted the influence of husbands and mothers-in-law in the decision to seek care. Many of the responses elicited may reflect what should be done, and not the actual behaviour of women.

Health care providers and community members alike claim there is routine use of antenatal care, though the number of visits is sometimes insufficient. Nearly two-thirds of women complete four antenatal visits, as recommended by the World Health Organization [1, 3, 18]. Two-thirds of women in Karnataka receive postnatal care [15]; however, as is true globally, most postnatal care is focussed on the neonate [19, 20] and the percent women whom receive maternal care after delivery is unknown. This may reflect a societal prioritization of caring for the infant and family over the mother. Case studies indicate that a lack transport is associated with maternal deaths in Karnataka [7]. Health care facilities are reportedly inadequate in Karnataka, with health personnel vacancies up to 52 %, weak information systems, lack of continuity of care, haphazard referral systems and distorted accountability mechanisms [7].

Efforts have been made to fill health service gaps with substantial success through free transport to facility, more community-based facilities, and financial subsidies. Further progress is, however, needed to avoid delays and ensure utilization of postpartum care.

As other studies have hypothesized the lack of sufficient progress on maternal mortality in India may be related to the strong emphasis on utilization of specific health services, namely ANC attendance and institutional deliveries [7]. Emphasis must equally be placed on other health services along the continuum of pregnancy and postpartum as well as the barriers to their use.

As with any study there are a number of strengths and limitations of this study and the methods used. The background of data collectors makes them well suited to conduct and record discussions in these communities; however their prior interaction with some interviewees may have led to altered responses. Participants may have been inclined to respond more favourably or have been hesitant to share concerns or faults. To minimize this social desirability bias, the study provided a clear explanation that all information is confidential, and would have no impact on their health care services or employment. The purposeful sampling approach may have resulted in a skewed population as community members were recruited through health staff and therefore those attending primary health centres were more likely to be chosen for participation; nevertheless, this approach was necessary due to budgetary and time constraints. Self-reported behaviours are often less reliable, yet is a common approach to obtaining information about health care utilization. Representation from many community and health system stakeholders provided a holistic view of these communities and breadth of beliefs and practices. The use of an *a priori* framework for analysis is limited by the deductive nature of the approach; however, inductive identification of themes was also included to supplement this framework. The responses elicited about use of maternity services are consistent with larger survey data from the area and with qualitative studies regarding the barriers to use of maternity care from other lower and middle income countries.

## Conclusions

Significant barriers exist to timely maternity and postpartum care, particularly related to transport, perceived quality of facilities, the cost of care, and the lack of recognition that a large proportion of maternal morbidity and mortality occurs in the postpartum period. These findings can be used for a number of purposes. Inadequacies were raised regarding quality of hospitals, these problems have been identified on a broader scale [9] and should be targeted by the health care system and administrators to encourage quality maternity care through training, hiring of additional staff and improvements to facility infrastructure. While maternity care use is nearly universal, timely care for complications and educating women and decision-makers about the importance of quality antenatal early in pregnancy and postpartum care should be targeted and strategies to overcome barriers should be developed through awareness raising programs. Such strategies should be incorporated into future health care worker training, community engagement activities, policy development and research. Although this study did not elicit information regarding disparities in utilization, others have found there is a continued need to overcome the barriers to utilization of maternity services among the most vulnerable groups, including culturally appropriate messages to target and engage them.

### Peer review

Peer review reports for this article can be found in Additional file 1.

## Additional file

Additional file 1: Peer review reports. (PDF 1014 kb)

## Abbreviations

ANC: Antenatal care
ANM: Auxiliary Nurse Midwives
ASHA: Accredited Social Health Activists
JNMC: Jawaharlal Nehru Medical College
NRHM: National Rural Health Mission
SNMC: S Nijalingappa Medical College
TBA: traditional birth attendant
UBC: University of British Columbia
