# Supplementary material for: Utilization of maternal health care services and their determinants in Karnataka State, India
Source: Reprod Health. 2016 Jun 8;13(Suppl 1):37. doi: 10.1186/s12978-016-0138-8 (PMC4943501; doi:10.1186/s12978-016-0138-8)
Supplement: Additional file 1: — Peer review reports. (PDF 1014 kb) [file 12978_2016_138_MOESM1_ESM.pdf]

Referee's comments to the authors– this sheet WILL be seen by the author(s) and published with the article

Title	**Utilization of maternal health care services and their determinants in Karnataka State, India**
Author(s)	Marianne Vidler, Umesh Ramadurg, Umesh Charantimath, Geetanjali Katageri, Chandrashekhar Karadiguddi, Diane Sawchuck, Rahat Qureshi, Shafik Dharamsi, Anjali Joshi, Richard Derman, Mrutyunjaya Bellad, Shivaprasad Goudar, Ashalata Mallapur for the Community Level Interventions for Pre-eclampsia (CLIP) India Feasibility Working Group
Referee's name	Jane Sandall

When assessing the work, please consider the following points, where applicable:

1. Is the question posed by the authors new and well defined?
2. Are the methods appropriate and well described, and are sufficient details provided to replicate the work?
3. Are the data sound and well controlled?
4. Does the manuscript adhere to the relevant standards for reporting and data deposition?
5. Are the discussion and conclusions well balanced and adequately supported by the data?
6. Do the title and abstract accurately convey what has been found?
7. Is the writing acceptable?

Please make your report as constructive and detailed as possible in your comments so that authors have the opportunity to overcome any serious deficiencies that you find and please also divide your comments into the following categories:

- Major Compulsory Revisions (which the author must respond to before a decision on publication can be reached)
- Minor Essential Revisions (such as missing labels on figures, or the wrong use of a term, which the author can be trusted to correct)
- Discretionary Revisions (which are recommendations for improvement but which the author can choose to ignore)

Where possible please supply references to substantiate your comments.

When referring to the manuscript please provide specific page and paragraph citations where appropriate.

General comments:

This is an important topic and unlike many studies of maternal health care utilization, which are often descriptive, the study design in this research has the potential to provide much more understanding of barriers and facilitators of maternal health care use. The design included a large number of focus groups with a substantial number of participants, representing a wide range of stakeholders including women, and interviews with stakeholders. In total 46 focus groups were held and 24 interviews. Data was transcribed and imported into NVivo. This is a lot of data for a qualitative study and the analysis is quite thin, with very few quotes to support the themes. This is a very carefully conducted study from a very large programme of research and having an understanding of why and how services are accessed and used is crucial to the successful aims set by planners and providers.

As the authors point out, they are perceived to be part of the wider study team and it is hard to avoid desirability response bias which is a major limitation. It might have been mitigated by some observation if this were logistically possible. The authors used an priori analysis framework. This is unusual with qualitative data. Please can the authors discuss the pros and cons of this approach and justify use. It may need to be discussed in study limitations section?

(continue on the next sheet)

Continued:

- Major Compulsory Revisions (which the author must respond to before a decision on publication can be reached)

Think about adding some more quotes to exemplify themes. These may need to be in an appendix due to word limit.

Consider what the learning is regarding how to avoid desirability response bias for future researchers. Locate findings more specifically in previous literature.

- Minor Essential Revisions (such as missing labels on figures, or the wrong use of a term, which the author can be trusted to correct)

  P6 l 85 how much compensation was given to participants?

- Discretionary Revisions (which are recommendations for improvement but which the author can choose to ignore)

Consider adding focus groups and qualitative methods and maternal health care utilization to key words.

P15 l 300. Do the authors mean ‘backward’ or do they mean low caste?

Utilization of maternal health care services and their determinants in Karnataka State, India

Marianne Vidler
Department of Obstetrics and Gynaecology, and the Child and Family Research Unit, University of British Columbia, Vancouver, British Columbia, Canada
Marianne.vidler@cw.bc.ca
*Corresponding author

Umesh Ramadurg
Department of Community Medicine, S Nijalingappa Medical College, Bagalkot, Karnataka, India
Umeshramadurg79@gmail.com

Umesh Charantimath
KLE University's Jawaharlal Nehru Medical College, Belgaum, Karnataka, India
Drumesh.charantimath@gmail.com

Geetanjali Katageri
Department of Obstetrics and Gynaecology, S Nijalingappa Medical College, Bagalkot, Karnataka, India
Geetanjali_mk@yahoo.co.in

Chandrashekhar Karadiguddi
KLE University's Jawaharlal Nehru Medical College, Belgaum, Karnataka, India
Karadiguddi.chandrashekhar@gmail.com

Diane Sawchuck
Department of Obstetrics and Gynaecology, and the Child and Family Research Unit, University of British Columbia, Vancouver, British Columbia, Canada
dsawchuck@shaw.ca

Rahat Qureshi
Division of Women and Child Health, Aga Khan University, Karachi, Sindh, Pakistan
Rahat.qureshi@aku.edu

Shafik Dharamsi
Department of Family Practice, Faculty of Medicine, University of British Columbia, Vancouver, British Columbia, Canada
Shafik.dharamsi@familymed.ubc.ca

Anjali Joshi,
KLE University's Jawaharlal Nehru Medical College, Belgaum, Karnataka, India
Dr.anjujoshi@yahoo.co.in

Peter von Dadelszen
Department of Obstetrics and Gynaecology, and the Child and Family Research Unit, University of British Columbia, Vancouver, British Columbia, Canada
pdadelszen@cw.bc.ca

Richard Derman

Department of Obstetrics, Christiana Care
RDerman@christianacare.org

Mrutyunjaya Bellad
KLE University's Jawaharlal Nehru Medical College, Belgaum, Karnataka, India
mbbellad@hotmail.com

Shivaprasad Goudar
KLE University's Jawaharlal Nehru Medical College, Belgaum, Karnataka, India
sgoudar@jnmc.edu

Ashalata Mallapur
Department of Obstetrics and Gynaecology, S Nijalingappa Medical College, Bagalkot, Karnataka, India
drashalatamallapur@gmail.com

for the Community Level Interventions for Pre-eclampsia (CLIP) India Feasibility Working Group
Department of Obstetrics and Gynaecology, and the Child and Family Research Unit, University of British Columbia, Vancouver, British Columbia, Canada
KLE University's Jawaharlal Nehru Medical College, Belgaum, Karnataka, India
Department of Obstetrics and Gynaecology, S Nijalingappa Medical College, Bagalkot, Karnataka, India

ABSTRACT

Background:

Karnataka State continues to have the highest rates of maternal mortality in south India at 144/100,000 live births, but lower than the national estimates of at 190-220/100,000 live births. Various barriers exist to timely and appropriate utilization of services during pregnancy, childbirth and postpartum. This study aimed to describe the patterns and determinants of routine and emergency maternal health care utilization in rural Karnataka State, India.

Methods:

Twenty three focus groups (332 respondents) and twelve individual interviews were conducted with relevant stakeholders in rural Karnataka in 2012-2013. Four focus groups were held with Auxiliary Nurse Midwives and Staff Nurses (N=48), 4 with Accredited Social Health Activists (N=53), 3 with community leaders (N=27), 2 with male decision-makers (N=19), 3 with female decision-makers (N=41), 6 with women of reproductive age (N=132), and one medical officers (N=12). One-to-one interviews were held with medical officers (N=2), private health care providers (N=2), senior health administrators (N=2), District health officers (N=2), and obstetricians (N=4). Local researchers familiar with the setting and language conducted focus groups and interviews. All discussions were audio recorded, transcribed, and translated for thematic analysis using NVivo 10.

Results:

Most women in the focus groups reported regular antenatal care attendance, for an average of four visits, and more often for high-risk pregnancies. Antenatal care was typically delivered at the periphery by non-specialised providers. Women reported they sought care if they experienced any danger signs. Postpartum care was reportedly rare, and mainly sought for the purpose of neonatal care. Factors that influenced women's care-seeking included their

limited autonomy, poor access to and funding for transport for non-emergent conditions, perceived poor quality of health care services and providers, and the costs of care.

Conclusions:

Rural south Indian communities reported regular use of health care services during pregnancy; however, barriers to access were prominent. Postpartum care continued to be rare. In spite of new government programmes and increased availability of maternity care services, some women still delayed or deferred accessing services. More efforts should be made to address the reported barriers that persist to maternity care services for women in rural Karnataka.

Trial Registration

NCT01911494

KEYWORDS

Pregnancy; Rural Population; Hypertension; Maternal Health Services; Maternal Mortality; Postnatal Care; Prenatal Care; Pregnancy, High Risk; India

BACKGROUND

Health care utilization overall, and for maternal health specifically, has improved in India. Much of the progress made has been attributed to the National Rural Health Mission (NRHM) that has increased the number of community health workers and community level facilities, and resulted in more institutional deliveries [1]. At present, virtually all pregnant women in Karnataka State make at least one antenatal care visit and nearly 95% deliver in facility [2]. However, utilization remains uneven and gaps remain in the provision of postnatal care, particularly in rural areas of India.

Explanatory factors for under-utilization of maternal care services are numerous, and include: young maternal age; religion (Muslims in Karnataka were less likely to receive the recommended four antenatal visits); poor education; unskilled occupation; poverty; low caste; parity (higher birth order were less likely to access antenatal services); lack of autonomy; poor familial support; lack of access to transport; and the high cost of care and when it is accessed, its poor quality (such as shortages of supplies and staff, mistreatment by staff, or poor training or limited experience of staff) [1,3, 4, 5, 6, 7, 8, 9]. These have been demonstrated overall and within Karnataka State, India specifically [1, 3].

As suboptimal levels of utilization are considered to contribute to poor maternal health, [1, 8, 10, 11], this study was designed to explore the patterns of maternal health care utilization in rural Karnataka State, India, as well as to identify the barriers that must be addressed to raise levels of that utilization with the ultimate goal of improving maternal and perinatal outcomes.

METHODS

Study setting

This qualitative study was conducted in two rural Districts of Karnataka State (Belgaum and Bagalkot) (Figure 1) where women have higher birth order, lower maternal age, and are more likely to be illiterate than women in other southern states [3]. These differences reflect variable political commitment to maternal health, poor implementation of the NRHM, and decentralization of services [12].

This study was conducted as part of a feasibility study for a cluster randomized controlled trial of a community-based treatment package for pre-eclampsia administered by community health care workers in Karnataka State (NCT01911494) [13]. This formative research focused

on exploration of the knowledge, attitudes and beliefs of community groups as well as facility assessments; the detailed methods are provided in [14].

Data collection

In brief, data were collected from January to March 2013 through focus group discussions and one-to-one interviews with community members and health care providers. Each focus group was assembled separately by type of respondent with the exception of Auxiliary Nurse Midwives (ANM) and staff nurses who participated together. All focus groups were conducted in the local language, Kannada, at primary health centres which are familiar to participants and have minimal distractions. All concerned senior health administrators, private consultants, and obstetricians at teaching institutes were recruited for in-depth interviews. All interviews were conducted in English. Interview participants were provided compensation for their time. Focus groups discussions lasted on average 60-90 minutes, while interviews were 45-60 minutes.

Focus group and interview guides were designed to focus on maternity care utilization and the challenges to it. *A priori*, the following thematic categories were to be explored for health care utilization for all of: routine antenatal care, pregnancy complications or danger signs, delivery, and postpartum care: decision-making power, availability of transport, quality of services and providers, financial constraints, and other sociocultural factors. All discussions were audio recorded, photographs and field notes were also taken. All recordings were reviewed to ensure accuracy; responses were then transcribed verbatim into English.

Analysis

With the *a priori* theme framework in mind (Figure 3), analysis began with a superficial reading to record initial thoughts, followed by a detailed review through face-to-face meetings including noting of any new emergent themes. Themes were developed and

grouped to reflect wider concepts; to provide insight into the subjective experiences of participants.

English interview and focus group transcripts, after initial analysis and de-identification, were sent to the study co-ordinating office, University of British Columbia (UBC), where they were reviewed and coded by the study qualitative research manager (MV). Once coded, all transcripts were returned to the relevant local research teams at Jawaharlal Nehru Medical College (JNMC), Belgaum and S Nijalingappa Medical College (SNMC), Bagalkot [illegible] confirm interpretation. Member checks were employed to ensure validity, reliability and accuracy of transcription and coding. At the time of review, any discrepancies were corrected, through review of original data and consensus was reached.

Ethical approval

This study was approved by the ethics review committees at the University of British Columbia, Vancouver Canada (H12-00132) and KLE University, Belgaum India (MDC/IECHSR/2011-12).

RESULTS

Participants

A total of twenty-three focus groups were held with relevant stakeholders: community health workers (ANMs and staff nurses) (n=48), Accredited Social Health Activists (ASHA) (n=53), community leaders (n=27), male (n=19) and female (n=41) decision-makers, women of reproductive age (n=132), and one with medical officers (n=12) (Table 2). Twelve interviews with two of each of the following stakeholders: medical officers (each of whom is responsible for one primary health centre that usually serves as the entry point to the health system), private consultants, senior health administrators, District health officers, and four obstetricians from tertiary level health care centres. Data saturation was judged to be

sufficiently met in the planned number of focus groups and interviews and no additional data collection was needed.

Respondent characteristics within each stakeholder group were relatively homogeneous but there were some between group differences (Table 2 & 3). All community health workers were actively providing care in the community at the time of data collection.

Maternal health care utilization

<u>Antenatal care</u>

The timing of the first antenatal care visit varies; according to providers there is now a trend to present earlier in gestation, by the end of the third month. Still, women described cultural beliefs that implore them to conceal pregnancies: "*It is a blind belief (superstition) [that it is best] not to disclose*" [ANM]. Identification of pregnancies may be limited by availability of pregnancy tests. Women do not systematically track their menstrual cycle, and have difficulty recalling their last menstrual period; as a result, asymptomatic women may not know they are pregnant. ASHAs attempt to track women of reproductive age in the community to identify their pregnancies early, but are challenged by women's concealment.

Once the pregnancy is disclosed, women generally attend antenatal care. "*During pregnancy we visit for check-up*" [woman of reproductive age]. The frequency of visits varies significantly between respondents, with women reporting an average of four antenatal care (ANC) visits. More visits are reportedly made near term and for those at high-risk of complications. Very few reported coming for fewer than three visits. This is adherent with and attributed to government programs and recommendations: "*Nowadays antenatal visits are more because of incentives*" [medical officer].

Antenatal care occurs at all levels of the health system (public and private); however, routine ANC is predominantly provided by community level health workers (ASHA, ANM,

Anganawadi worker, staff nurse) in the home or government run centres (sub-centre, primary health centre, community health centre). Anganawadi workers provide the most basic health services in rural Indian communities. In these communities, there is no longer regular use of Dais (traditional birth attendants): "*No Dais working now, all that has decreased now*" [ANM/staff nurses]. Consistent with their recommended roles, ASHAs are seen as facilitators for care, providing prevention and education, while staff nurses and medical officers provide testing and treatment, as appropriate. Similarly, though many women use folk remedies in pregnancy, the use of traditional providers is rare – "*In older times there were many such people [traditional healers]*".

<u>Complications</u>

There was significant discussion regarding care seeking for danger signs and pregnancy complications. It was widely agreed that care should be sought if a woman is at high-risk of complications or if she displayed danger signs, such as eclampsia (see below). The responses focused on what is recommended, which may not reflect the reality of practice. Almost all women in the area delivered institutionally at the time of this discussion, and thus reported using services for complications or emergencies. One woman described her feelings regarding accessing services for emergencies: "*The women do not have any problem in going to hospital during emergency…If they are not feeling alright then only they go to the hospital*" [woman of reproductive age]. Generally, if complications are perceived to be minor, women access care at the primary health centre; however, if they are perceived as more serious, care is sought at a higher-level facility where specialists are available. There was particularly rich discussion related to pre-eclampsia and eclampsia as this was a topic of interest for researchers and probed directly: "*I had fits in the home so I went to the hospital*"

[woman of reproductive age]. Bleeding and the absence of fetal movements were also commonly mentioned as reasons for care seeking at the higher facility.

Delivery

Many women stated that they visit the hospital at the time of delivery, supported by the high rates of facility deliveries. Women attributed facility birth to government financial and material incentives, such as small remuneration and basic items (Madilu kit) for care of the newborn. An ANM/staff nurses in Bagalkot described the influence of these demand side initiatives: "*Women don't deliver at home now at least for the sake of Madilu Kit*" [ANM/staff nurses].

Postpartum

Few respondents mentioned postpartum health care utilization. When they did, care was usually sought for newborn care or feeding difficulties: "…*if the baby cannot breastfeed* [ANM/staff nurse]. Some community health care providers stated that women rarely access services after delivery until the infant requires immunizations.

How providers encourage women to seek care

Health care providers have adopted a number of strategies to encourage women to better access services. Some providers stated that they offer to: accompany women to referral facilities, provide services in the home, and supply financial assistance or even free services. In addition, providers stated that they focus on education throughout pregnancy to encourage appropriate and timely use of facility services, particularly for delivery. The education was described as informing families, particularly husbands and mothers-in-law, of available facilities and services, possible pregnancy complications, and the importance of antenatal

care. One medical officer described the components of this education: "*If you get a check-up, what will happen, if you don't get a check-up, that we will tell and convince and will make them to come to the hospital. Calling the mothers-in-law and whatever relations they will be having call them and explain the risks and everything and what happens in the regular ANC check up and take iron tablets*" [medical officer].

Determinants of health care utilization in pregnancy and postpartum

<u>Decision-making power</u>

Respondents confirmed that the autonomy of women in patriarchal Indian communities remains limited. Respondents almost unanimously claimed that women have restricted decision-making power concerning their own health, and few mentioned that this may be changing: "*Nowadays [women] take their own decisions*" [female decision-maker]. Mothers-in-law and husbands hold the decision-making power, and they often resist facility-based care. Stated reasons for this included the family's history of successful home births: "*I delivered 10 to 12 times without any problems, why she should go to the hospital for delivery?*" [ANM/staff nurse]. As a result, women who are home alone must wait for a designated decision-maker before appropriate action can be considered. This lack of familial support was reported to be widespread and to prevent or delay care-seeking. At times, neighbours may stand in if the household decision-maker is unavailable.

<u>Availability of transport</u>

Communities in Karnataka reported awareness of '108' - the robust ambulance system available free of charge for emergencies: *"If we have to go to Bagalkot they provide ambulance free of cost, previously it was not good, now it is good"* [community leader]. The challenges expressed related to transport for routine care. Women may need to hire private

vehicles, but some villages do not have access to these as backup and some families cannot afford this. The price of private transport varies depending on the mode, time of day and distance, some state it can be as little as 100 Rupees (equivalent to $1.50 USD) and as high as 3,000 Rupees (equivalent to $47 USD). Many respondents stated that at the community level, funds are available for transport for those below the poverty line. Aside from availability and cost, access was described to be further complicated by the terrain, with narrow roads and villages at long distances from urban centres. These were stated to be barriers to seeking transport and care, particularly at night.

Quality of services and providers

Most respondents stated they were satisfied with the quality of services at facilities: “*All are happy about the quality”* [male decision-maker]. However, some complained about poor quality of services and treatment as a barrier to using care. Specific issues raised included lack of sufficient staffing in facilities and inadequate infrastructure, such as unreliable power and poor level of cleanliness: “*We need more rooms, good water, consistent electricity, and also tea and food should be given”* [woman of reproductive age]. Also, facilities are reportedly short of the necessary medical equipment, materials, lab services, blood, and medications.

The majority of participants stated that they had been well treated by health care staff: “*Our health care providers speak nicely and they see us calmly and solve any doubt we have, we are happy for that”* [woman of reproductive age]. Previous experiences were mentioned as influencing a woman’s service utilization in a subsequent pregnancy: “*Wherever they have faith, they will go; it is dependent on their belief. They would have gone repeatedly before and they have felt good at that time”* [community leader]. Trust and respect are essential

qualities in a health care provider, according to women of reproductive age. Obstetricians themselves claim when women have trust in the doctor they will come to facility.

Financial constraints

Respondents indicated that the costs of health care inhibit women's ability to access health care services: "*Fear of cost at facility makes them think twice*" [ANM/staff nurse]. These costs are related to transport, medications, services, lost wages, and food. Respondents reported that the cost of delivery ranges from 500Rs (equivalent to $8 USD) up to 100,000Rs (equivalent to $1,570 USD). The cost of delivery largely depends on the type of facility (government vs private) and the services provided (mode of delivery and associated complications). The community may provide financial support to women in need through women's groups (Stree Shakti Sangh), Gram Panchayat (local self-government), and government programs. Those who qualify as below the poverty line are provided these subsidies and additional benefits to encourage appropriate care-seeking.

DISCUSSION

Several underlying factors have been identified here that play important roles in health care seeking: autonomy, access to transport, quality of services, incentive-based programs and poverty. Efforts have been made to fill health service coverage gaps with substantial success through free transport to facility, more community-based facilities, and financial subsidies for those in need. Further progress is, however, needed to ensure timely care and postpartum care.

Many of the responses elicited may reflect what should be done, and not the actual behaviour of women. Health care providers and community members alike claim there is frequent and appropriately early use of services for routine antenatal care, though the number

of visits is sometimes insufficient, and there are similarly high rates of institutional delivery in the study area.

One of the limitations of this study is that detailed information was not elicited about complications, their severity and timely care seeking. Severe complications are relatively rare, and therefore may not have had participants who experienced such complications. Postpartum care is reportedly infrequent; the care seeking at this time is typically related to the infant. This may reflect a societal prioritization of caring for the infant and family over the mother. As other studies have hypothesized the lack of sufficient progress on maternal mortality in India may be related to the strong emphasis on utilization of specific health services, namely ANC attendance and institutional deliveries [7]. Emphasis must equally be placed on other health services along the continuum of pregnancy and postpartum as well as the barriers to their use. Overall, the community does not perceive pregnancy to be an inherently dangerous experience.

As with any study there are a number of strengths and limitations of these findings and the methods used. The background of data collectors makes them well suited to conduct and record discussions in these communities. Nevertheless, as some facilitators had existing relationships with participants this may have introduced bias. Some respondents may have been inclined to respond more favourably or have been hesitant to share concerns or faults. To minimize this bias, the study provided a clear explanation that all information is confidential, and would have no impact on their health care services. The use of self-reported behaviours may be more or less reliable, but is a common approach to obtaining information about health care utilization. The responses elicited about use of maternity services are consistent with larger survey data from the area and with qualitative studies regarding the barriers to use of maternity care from other lower and middle income countries.

Nearly only two-thirds of women complete four antenatal visits, as recommended by the World Health Organization [1, 3, 15]. Most women attending ANC in Karnataka were seen by doctors (79%) or Auxiliary Nurse Midwives (10%) [16]. It has been shown that women who reside in rural areas present later in gestation [5, 16]. Fifty-seven percent of women currently deliver in hospitals or primary health centres while 36% continue to deliver at home [1]. Women from backward or general caste, below the poverty line, and without previous ANC are less likely to deliver in facility [1]. Roughly half of women deliver with assistance of a skilled birth attendant during delivery, while 26% continue to have a traditional birth attendant (TBA) and 8% deliver with only a family member [1]. TBAs continue to play a significant role in these communities [1, 8]. Two-thirds of women in Karnataka receive postnatal care [16]; however, as is true globally, most postnatal care is focussed on the neonate [17,18] and the percent women whom receive maternal care after delivery is unknown.

Women's autonomy (decision-making power related to her own health, buying household items, going to stay with family, and access to funds) is low in India which influences utilization of ANC, delivery and postpartum examination [19, 20]. Women's autonomy in India has been found to be related to her husband's position within the family and family composition [20]. Proximity and ties to the natal family are also associated with age of marriage, autonomy and ultimately health care utilization [20]. Rural residence is associated with decreased use of skilled attendance at delivery [8]. Case studies indicate that a lack transport is associated with maternal deaths in Karnataka [7]. Quality of care is often inadequate in Karnataka, with health personnel vacancies up to 52%, weak information systems, lack of continuity of care, haphazard referral systems and distorted accountability mechanisms [7]. Poverty is strongly correlated with lower rates of institutional delivery in Karnataka [1, 7]. Karnataka is in the top ten states with scheduled castes or tribes. These

women are twice as likely to deliver outside a facility and have poorest health outcomes overall [1, 3]. Lower health care utilization may be related to perceived stigma [1]. Age and parity also influence maternity care use. Younger (under the age of 18) and primiparous women are less likely to delivery in health care facilities services [3, 4], women with higher birth orders (2+), who are older and last gave birth a longer time ago are less likely to access ANC and facilities for delivery [3]. Women's education was directly associated with ANC use, as well as the number of ANC visits, and institutional delivery in Karnataka [3, 4, 5, 6]. Women with husbands in the business sector utilize maternity care services more often than wives of farmers [4]. The association between religion and maternity care is less clear and confounded with socio-economic and other factors [3, 6].

CONCLUSIONS

Significant barriers exist to timely maternity and postpartum care use, particularly related to transport, perceived quality of care and cost, and the lack of recognition that a large proportion of maternal morbidity and mortality occurs in the postpartum period. These findings can be used for a number of purposes. Inadequacies were raised regarding quality of care: number of staff, facilities, and treatment by providers. These problems have been identified on a broader scale [9] and should be targeted by the health care system and administrators to encourage quality maternity care through proper training both for knowledge and skills that can improve maternal and neonatal survival. While maternity care use is nearly universal, timely care for complications and educating women and decision-makers about the importance of quality antenatal and postpartum care should be targeted and strategies to overcome barriers should be developed through proper awareness programs. Such strategies should be incorporated into future health care worker training, community engagement activities, policy development and future research. Although this study did not

elicit information regarding disparities in utilization, others have found there is a continued need to overcome the barriers to utilization of maternity services among the most vulnerable groups, including culturally appropriate messages to target and engage them.

LIST OF ABBREVIATIONS

National Rural Health Mission (NRHM); University of British Columbia (UBC); Jawaharlal Nehru Medical College (JNMC); S Nijalingappa Medical College (SNMC); Auxiliary Nurse Midwives (ANM); Accredited Social Health Activists (ASHA); Antenatal care (ANC); Traditional Birth Attendant (TBA)

COMPETING INTERSTS

The authors declare they have no competing interests

AUTHOR'S CONTRIBUTIONS

MV drafted the manuscript and conducted qualitative analysis. DS and RQ contributed to the conception and design of the study. PvD, SG, MB, RD, and AM provided critically important revision of the manuscript and interpretation of the data; in addition they provided final approvals. SD provided critical intellectual input to manuscript development. UC, GK, UR, AJ and CK oversaw all data acquisition and analysis, as well as involvement in revision of the manuscript.

ACKNOWLEDGMENTS

This work is part of the University of British Columbia PRE-EMPT (Pre-eclampsia/Eclampsia, Monitoring, Prevention and Treatment) initiative supported by the Bill & Melinda Gates Foundation. We gratefully acknowledge contributions of the Community Level Interventions for Pre-eclampsia (CLIP) India Feasibility Working Group: Shashidhar

Bannale, Beth Payne, Zulfiqar Bhutta, Laura Magee, Chirag Kariya, Sharla Drebit, Sheela Naik, Sangmesh Rakkareddi, and Amit Revankar. We also acknowledge the support of KLE University, JN Medical College, SN Medical College, University of British Columbia, Government of Karnataka, the district health administration of Belgaum and Bagalkot Districts, the local medical officers, staff nurses, auxiliary nurse midwives, accredited social health activists, and other health centre staff and community leaders for their participation and their efforts in motivating community participation. We thank the research office staff for assisting in data translation and transcription. Finally, a special thanks to all the focus group and interview participants.

REFERENCES

[1] ..., N. Kassebaum, A. Bertozzi-Villa, M. Coggeshall, K. Shackelford, C. Steiner, K. Heuton, D. Gonzalez-Medina, R. Barber, C. Huynh, D. Dicker, T. Templin, T. Wolock, A. Ozgoren, F. Abd-Allah, S. Abera, I. Abubakar, T. Achoki, A. Adelekan, Z. Ademi, A. Adou, J. Adsuar, E. Agardh, D. Akena, D. Alasfoor, Z. Alemu, R. Alfonso-Cristancho, S. Alhabib, R. Ali, M. Al Kahbouri, F. Alla, P. Allen, M. AlMazroa, U. Alsharif, E. Alvarez, N. Alvis-Guzman, A. Amankwaa, A. Amare, H. Amini, W. Ammar, C. Antonio, P. Anwari, J. Arnlov, V. Arsenijevic, A. Artaman, M. Asad, R. Asghar, R. Assadi, L. Atkins, A. Badawi, K. Balakrishnan, A. Basu, S. Basu, J. Beardsley, N. Bedi, T. Bekele, M. Bell, E. Bernabe, T. Beyene, Z. Bhutta, A. Bin Abdulhak, J. Blore, B. Basara, D. Bose, N. Breitborde, R. Cardenas, C. Castaneda-Orjuela, R. Castro, F. Catala-Lopez, A. Cavlin, J. Chang, X. Che, C. Christophi, S. Chugh, M. Cirillo, S. Colquhoun, L. Cooper, C. Cooper, I. Leite, L. Dandona, R. Dandona, A. Davis, A. Dayama, L. Degenhardt, D. De Leo, B. del Pozo-Cruz, K. Deribe, M. Dessalegn, G. deVeber, S. Dharmaratne, U. Dilmen, E. Ding, R. Dorrington, T. Driscoll, S. Ermakov, A. Esteghamati, E. Faraon, F. Farzadfar, M. Felicio, S. Fereshtehnejad, G. de Lima, Akademin Hälsa och samhälle, Medicinsk vetenskap and Högskolan Dalarna. Global, regional, and national levels and causes of maternal mortality during 1990-2013: A systematic analysis for the global burden of disease study 2013. *Lancet 384(9947),* pp. 980-1004. 2014. . DOI: 10.1016/S0140-6736(14)60696-6.

[1] P. C. Adamson, K. Krupp, B. Niranjankumar, A. H. Freeman, M. Khan and P. Madhivanan. Are marginalized women being left behind? A population-based study of institutional deliveries in karnataka, india. *BMC Public Health 12(1),* pp. 30-30. 2012; 2011. . DOI: 10.1186/1471-2458-12-30.

[2] Goudar SS, Goco N, Somannavar MS, Vernekar SS, Mallapur AA, Janet L. Moore JL, Wallace DD, Sloan NL, Derman RJ, Patel A, Hibberd PL, Koso-Thomas M, Goldenberg RL. Institutional deliveries and perinatal and neonatal mortality in Southern and Central India. BMC Reproductive Health (In Press)

[3] K. Navaneetham and A. Dharmalingam, "Utilization of maternal health care services in Southern India," *Soc. Sci. Med.,* vol. 55, pp. 1849-1869, 11, 2002.

[4] A. Srivastava, S. Mahmood, P. Mishra and V. Shrotriya. Correlates of maternal health care utilization in rohilkhand region, india. *Annals of Medical and Health Sciences Research 4(3),* pp. 417. 2014. . DOI: 10.4103/2141-9248.133471.

[5] J. C. Bhatia and J. Cleland. Determinants of maternal care in a region of south india. *Health Transition Review 5(2),* pp. 127-142. 1995.

[6] S. S. Bloom, D. Wypij and M. d. Gupta. Dimensions of women's autonomy and the influence on maternal health care utilization in a north indian city. *Demography 38(1),* pp. 67-78. 2001. . DOI: 10.1353/dem.2001.0001.

[7] A. George. Persistence of high maternal mortality in koppal district, karnataka, india: Observed service delivery constraints. *Reprod. Health Matters 15(30),* pp. 91-102. 2007. . DOI: 10.1016/S0968-8080(07)30318-2.

[8] C. Kumar, P. K. Singh and R. K. Rai. Coverage gap in maternal and child health services in india: Assessing trends and regional deprivation during 1992-2006. *Journal of Public Health (Oxford, England) 35(4),* pp. 598. 2013.

[9] O. Pasha, E. McClure, L. Wright, S. Saleem, S. Goudar, E. Chomba, A. Patel, F. Esamai, A. Garces, F. Althabe, B. Kodkany, H. Mabeya, A. Manasyan, W. Carlo, R. Derman, P. Hibberd, E. Liechty, N. Krebs, K. Hambidge, P. Buekens, J. Moore, A. Jobe, M. Koso-Thomas, D. Wallace, S. Stalls, R. Goldenberg and EMONC Trial Investigators. A combined community- and facility-based approach to improve pregnancy outcomes in low-resource settings: A global network cluster randomized trial. *Bmc Medicine 11*pp. 215. 2013. . DOI: 10.1186/1741-7015-11-215.

[10] S. Thaddeus and D. Maine. Too far to walk: Maternal mortality in context. *Soc. Sci. Med. 38(8),* pp. 1091-1110. 1994. . DOI: 10.1016/0277-9536(94)90226-7.

[11] F. Bustreo, L. Say, M. Koblinsky, T. Pullum, M. Temmerman and A. Pablos-Mendez. Ending preventable maternal deaths: The time is now. *Lancet Global Health 1(4),* pp. E176-E177. 2013. . DOI: 10.1016/S2214-109X(13)70059-7.

[12] S. L. Smith, "Political contexts and maternal health policy: Insights from a comparison of south Indian states," *Soc. Sci. Med.,* vol. 100, pp. 46-53, 1, 2014.

[13] P. von Dadelszen, M.A. Magee, B.A. Payne, Z. Bhutta, “The CLIP (Community-Level Interventions for Pre-eclampsia) cluster randomized controlled trial,” *The Lancet* [*in press*]

[14] A. Khowaja, R. Qureshi, D. Sawchuck, O. Oladapo, O. Adetoro and et al., "The feasibility of community level interventions for pre-eclampsia in South Asia and Sub-Saharan Africa: A mixed-methods design," *BioMed Central Reproductive Health,* 2015. *[in press]*

[15] World Health Organization, "Trends in Maternal Mortality: 1990 to 2013," 2014.

[16] International Institute for Population Sciences (IIPS) and Macro International, "National Family Health Survey (NFHS-3), 2005–06: India ," *International Institute for Population Sciences,* vol. 1, pp. 195, 2007.

[17] X. Li, J. Fortney, M. Kotelchuck and L. Glover, "The postpartum period: the key to maternal mortality," *International Journal of Gynecology and Obstetrics,* vol. 54, 1996.

[18] World Health Organization, "WHO technical consultation on postpartum and postnatal care," 2010

[19] R. Mistry, O. Galal and M. Lu. Women's autonomy and pregnancy care in rural india: A contextual analysis. *Soc. Sci. Med. 69(6),* pp. 926-933. 2009. . DOI: 10.1016/j.socscimed.2009.07.008.

FIGURES

Figure 1. Map of study sites, Karnataka State, India

Figure 2. Focus group discussion with male decision-makers in rural Karnataka

Figure 3. Thematic analysis structure

TABLES

Table 1. Site characteristics

Table 2. Characteristics of focus group participants

Table 3. Characteristics of interview participants

TABLES

Table 1 Site characteristics

	INDIA	SOUTH INDIA	KARNATAKA
**SITE CHARACTERISTICS**			
POPULATION	1,236,687		61,130,704 [5]
# STATES	35		
DOMINANT RELIGION	Hindu [3]	Hindu [3]	Hindu [3]
WOMEN'S LITERACY	55% [3]	68% [3]	58% [3]
EMPLOYMENT	36% currently employed [3]	41% currently employed [3]	40% currently employed [3]
RURAL /URBAN	32% urban [1]		39% urban [5]
FERTILITY RATE	2.8 [3]	1.9 [3]	2.1 [3]
MATERNAL MORTALITY RATIO	178 per 100,000 live births [2]	105 per 100,000 live births [1]	144 per 100,000 live births [1]
**MATERNAL HEALTH CARE UTILIZATION**			
ANY ANC	76.4% 3	94% [3]	89% [3]
≥4 ANC	48% [3]	89% [3] *(3+)*	76% [3] *(3+)*
FACILITY DELIVERY (%)	39% [3]	79% [3]	65% [3]
SKILLED ATTENDANT AT DELIVERY	47% [3]	84% [3]	70% [3]

[1] *World Health Organization Country Profile: India 2012*
[2] *Office of the Registrar of India, 2013*
[3] *Demographic Health Survey 2013*
[4] *National Rural Health Mission, Census 2011*
[5] *Rural Health Statistics in India 2012*

Table 2 Characteristics of focus group participants

#	N participants	Age (yr)<br>Median [range]	Occupation<br>1 Housewife<br>2 Labourer<br>3 Employee<br>4 Self-employed<br>5 Other	Child <5yr	Pregnant	Education<br>1 No formal schooling, cannot read or write<br>2 No formal schooling, can read and write<br>3 Primary school incomplete<br>4 Primary school complete<br>5 Secondary school incomplete<br>6 Secondary school complete<br>7 Pre-university incomplete<br>8 Pre-university complete<br>9 University incomplete<br>10 University complete<br>11 Postgraduate<br>12 Don't know	Relationship to woman<br>1 Husband<br>2 Father<br>3 Father-in-law<br>4 Mother-in-law<br>5 Mother<br>6 Other
**Community Leaders**							
1	7	36<br>[31,48]	1 = (N=1)<br>2 = (N=6)	*Not asked*	*Not asked*	1 = (N=1)<br>6 = (N=1)<br>8 = (N=2)<br>10 = (N=3)	*Not asked*
2	10	36<br>[24,51]	1 = (N=3)<br>4 = (N=3)<br>5 = (N=4)	*Not asked*	*Not asked*	1 = (N=1)<br>3 = (N=1)<br>4 = (N=1)<br>5 = (N=1)<br>6 =(N=3)<br>8 = (N=1)<br>9 = (N=1)<br>10 = (N=1)	*Not asked*
3	10	*Not known*	*Not known*	*Not asked*	*Not asked*	*Not known*	*Not asked*
**Male Decision-Makers**							
1	8	26<br>[18,57]	2 = (N=4)<br>3 = (N=1)<br>4 = (N=1)<br>5= (N=2)	*Not Applicable*	*Not Applicable*	1 = (N=4)<br>3 = (N=2)<br>6 = (N=2)	1 = (N=4)<br>3 = (N= 2)<br>6 = (N=2)
2	11	49<br>[33,59]	3 = (N=2)<br>5 = (N=9)	*Not Applicable*	*Not Applicable*	3 = (N=4)<br>5 = (N=1)<br>6 = (N=1)<br>10 = (N=1)<br>12 = (N=4)	2 = (N=4)<br>3 = (N=3)<br>6 = (N=4)
**Female Decision-Makers**							
1	10	45<br>[30,60]	1 = (N=9)<br>2 = (N=1)	*Not Applicable*	*Not Applicable*	1 = (N=8)<br>4 = (N=2)	4 = (N=6)<br>5 = (N=2)<br>6 = (N=2)
2	18	45<br>[28,65]	1 = (N=5)<br>3 = (N=1)<br>4 = (N=1)<br>5 = (N=11)	*Not Applicable*	*Not Applicable*	1 = (N=3)<br>2 = (N=1)<br>3 = (N=1)<br>12 = (N=13)	*Not Known*
3	13	48	1 = (N=13)	*Not*	*Not*	3 = (N=1)	4 = (N=7)

		[30,65]		*Applicable*	*Applicable*	5 = (N=1)<br>12 = (N=11)	5 = (N=1)<br>6 (N=5)
**Women of Reproductive Age**							
1	55	*Not Known*	*Not Known*	*Not Known*	*Not Known*	*Not Known*	*Not Known*
2	16	25<br>[20,30]	1 = (N=16)	56%	75%	3 = (N=3)<br>4 = (N=1)<br>5 = (N=1)<br>6 = (N=2)<br>8 = (N=2)<br>10 = (N=1)<br>12 = (N=6)	*Not Applicable*
3	14	23<br>[18,30]	1 = (N=9)<br>2 = (N=5)	36%	86%	1 = (N=3)<br>3 = (N=3)<br>4 = (N=1)<br>5 = (N=1)<br>6 = (N=3)<br>8 = (N=2)<br>10 = (N=1)	*Not Applicable*
4	17	*Not Known*	1 = (N=17)	88%	100%	*Not Known*	*Not Applicable*
5	14	22<br>[18,58]	1 = (N=12)<br>3 = (N=2)	71%	50%	3 = (N=3)<br>5 = (N=3)<br>6 = (N=3)<br>10 = (N=2)<br>12 = (N=3)	*Not Applicable*
6	16	20<br>[19,26]	1 = (N=16)	69%	63%	3 = (N=7)<br>4 = (N=4)<br>5 = (N=1)<br>6 = (N=3)<br>8 = (N=1)	*Not Applicable*
**Auxiliary Nurse Midwives and Staff Nurses**							
1	8	36<br>[24,56]	*Not Known*	*Not Asked*	*Not Asked*	6 = (N=3)<br>8 = (N=1)<br>10 = (N=1)<br>11 = (N=3)	*Not Applicable*
2	9	41<br>[23,58]	5 = (N=9)	*Not Asked*	*Not Asked*	6 = (N=2)<br>7 = (N=1)<br>8 = (N=3)<br>10 = (N=3)	*Not Applicable*
3	19	39<br>[25,58]	SN (N=7)<br>ANM (N=10)	*Not Asked*	*Not Asked*	*Not Known*	*Not Applicable*
4	12	30<br>[24,53]	SN (N=4)<br>ANM (N=4)	*Not Asked*	*Not Asked*	*Not Known*	*Not Applicable*
**Accredited Social Health Activists**							
1	10	32<br>[26,36]	*Not Known*	*Not Asked*	*Not Asked*	*Not Known*	*Not Applicable*
2	11	*Not Known*	*Not Known*	*Not Asked*	*Not Asked*	*Not Known*	*Not Applicable*
3	15	*Not Known*	ASHA (N=15)	*Not Asked*	*Not Asked*	*Not Known*	*Not Applicable*
4	17	33<br>[21,44]	ASHA (N=17)	*Not Asked*	*Not Asked*	*Not Known*	*Not Applicable*
**Medical Officers**							

1	15	*Not Known*	*Not Asked*	*Not Asked*	*Not Asked*	*Not Asked*	*Not Applicable*

Table 3. Characteristics of interview participants

#	Stakeholder	Training	Level of Care	Pregnancies / Week	Catchment Pop	Pre-eclampsia /12 Months
1	Medical Officer	MBBS	Primary	50-60	19,000	5-6
2	Medical Officer	MBBS	Primary	10-15	35,000	20
3	Private Practitioner	MD OBG	Tertiary	40-50	200,000	50-100
4	Private Practitioner	MBBS & MD OBG	Tertiary	280-300	300-400,000	20-25
5	Senior Health Administrator	MS General Surgery	--	--	1,950,000	--
6	Senior Health Administrator	MBBS & PG in OBG	--	--	4,800,000	--
7	District Health Officer	Speciality DGO	Secondary	200-250	25,000	12-25
8	District Health Officer	MBBS & Diploma OBG	Secondary	50-60	270,000	200-220
9	Obstetrician	MD OBG	Tertiary	250	Unknown	80-100
10	Obstetrician	MBBS & DGO & MD	Tertiary	200-300	300,000	500+
11	Obstetrician	MBBS & Diploma OBG	Tertiary	45	Unknown	150-200
12	Obstetrician	MD OBG	Tertiary	--	800-900,000	15-17%

Figure 1

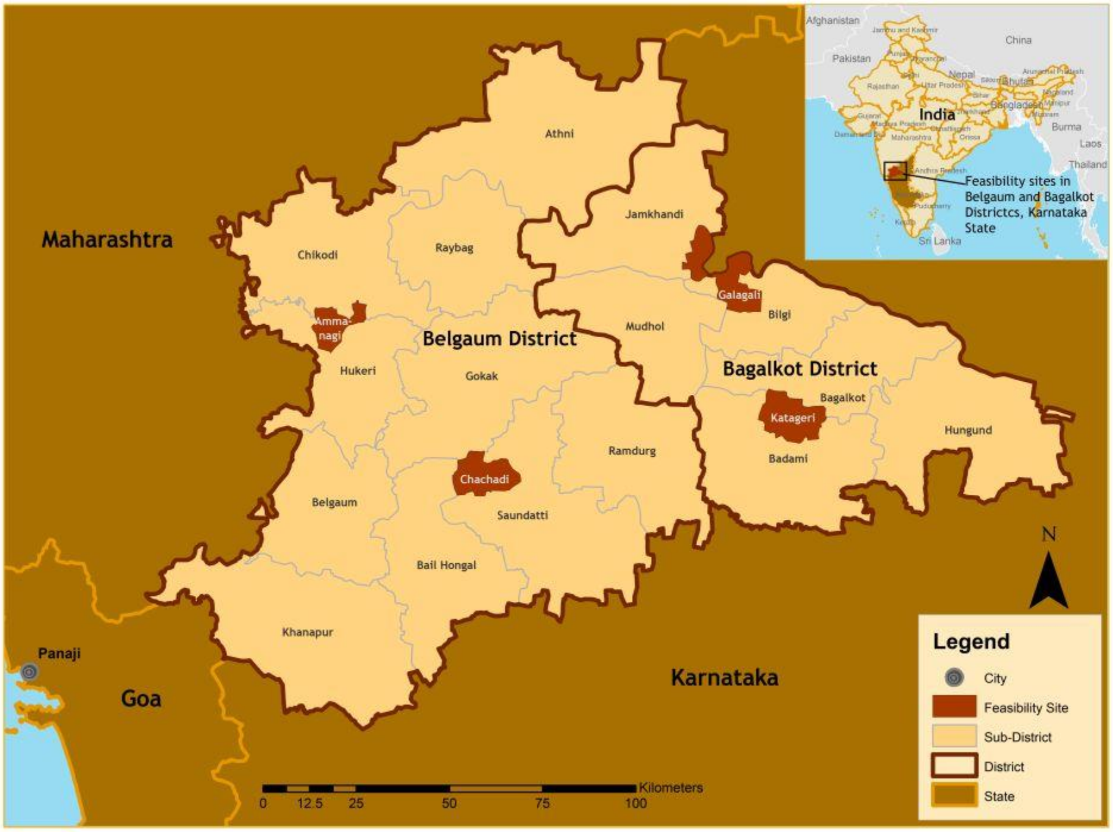
Afghanistan
Pakistan
China
Nepal
India
Rajasthan
Uttar Pradesh
Bihar
Bangladesh
Gujarat
Maharashtra
Orissa
Andhra Pradesh
Burma
Laos
Thailand
Sri Lanka
Feasibility sites in Belgaum and Bagalkot Districtcs, Karnataka State
Maharashtra
Athni
Jamkhandi
Chikodi
Raybag
Galagali
Amma-nagi
Mudhol
Bilgi
Belgaum District
Bagalkot District
Hukeri
Gokak
Bagalkot
Katageri
Hungund
Ramdurg
Badami
Chachadi
Belgaum
Saundatti
Bail Hongal
Khanapur
Panaji
Goa
Karnataka
N
Legend
City
Feasibility Site
Sub-District
District
State
0
12.5
25
50
75
100
Kilometers

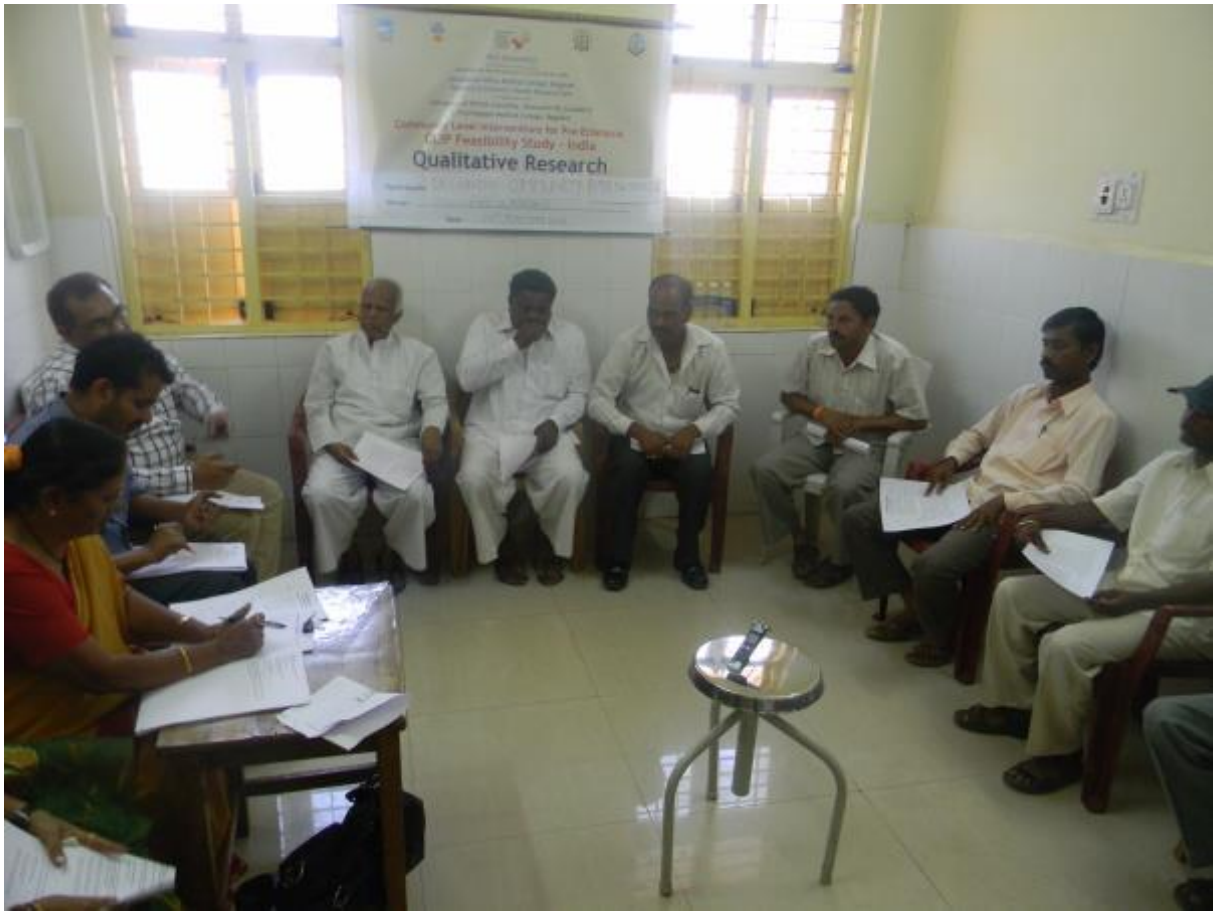
Qualitative Research

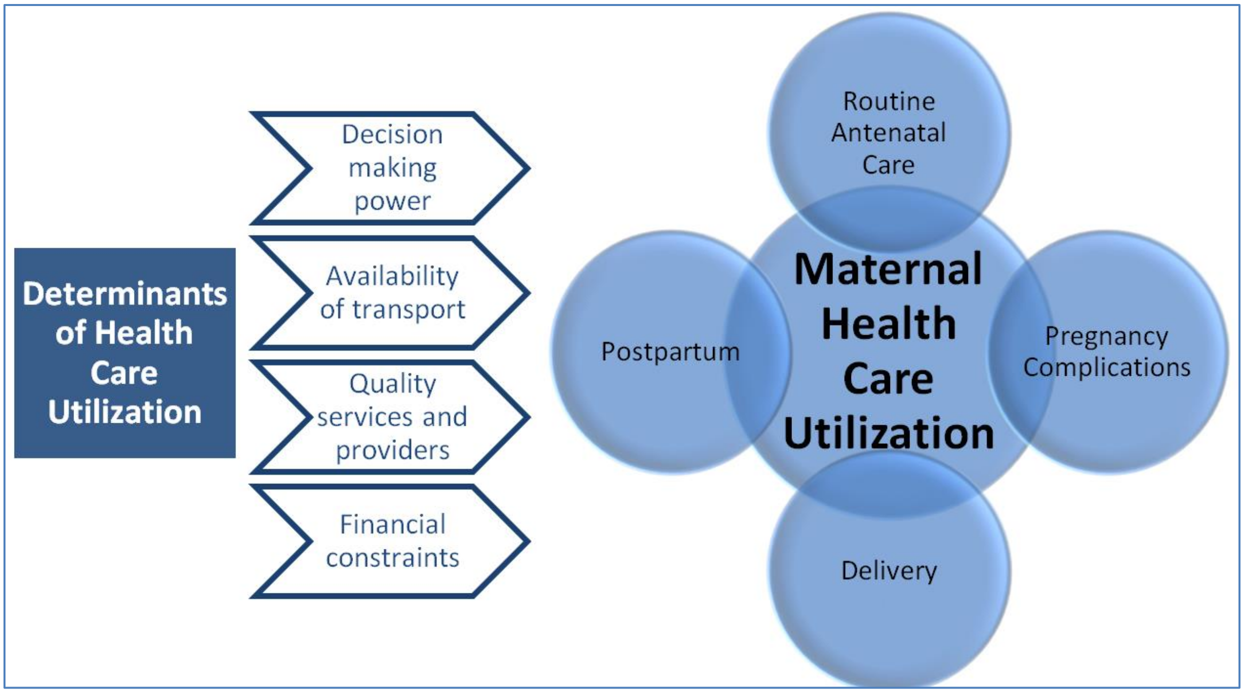
Determinants of Health Care Utilization
Decision making power
Availability of transport
Quality services and providers
Financial constraints
Routine Antenatal Care
Postpartum
Maternal Health Care Utilization
Pregnancy Complications
Delivery
